# A Case of Calcification of a Widely Exposed Pulp

**Published:** 1896-08

**Authors:** Charles S. Tomes


### Abstracts and Translations.

### A CASE OF CALCIFICATION OF A WIDELY EXPOSED PULP.

BY MR. CHARLES S. TOMES.

The specimen to which I would call your attention is one so remarkable, and also so instructive from several points of view, that it will fully repay somewhat close examination.

The tooth is one which, having been broken right across its pulp-cavity, has, nevertheless succeeded in repairing the damage and sealing it up again, an event of such rarity that I only know of one other recorded case,—namely, that to be found figured in my father's "Dental Surgery," third edition, page 341. Had my specimen been quite unique I should have hesitated to cut it up for microscopic examination, and then should have missed what, to my mind, are the points of greatest interest it has to show. The tooth is either a lower wisdom or a somewhat small second molar, the loss of the neighboring teeth rendering its exact determination impossible.

The patient is a servant of a patient of mine, and was brought to me on account of very severe but intermittent pain of a neuralgic type, recurring most days, but not every day. I found a tiny opening in the gum, which was otherwise of healthy appearance. A probe introduced disclosed the presence of what felt like a complete but rather rough-surfaced tooth. The history was that some three years previously an attempt had been made to extract a tooth in this situation, but that it had been broken off. It was exquisitely tender, and occasionally very painful for a long time afterwards, but it gradually got better and healed over, but it never remained absolutely comfortable for long together, although the severe paroxysmal pain which brought her to me was only of a few weeks' standing. With the aid of cocaine I reflected the gum from over it, and subsequently had no difficulty in removing it with an elevator. I then found that it had been transversely fractured a little way above its neck, and that what should have been an open pulp-cavity was occupied by a cauliflower-shaped mass of shining polished ivory projecting above the original surface of fracture and overflowing onto it. By cutting it carefully with a hair saw I was able to get four good longitudinal sections, and found that the pulp was not wholly calcified, but that a residue of the pulp-chamber was still occupied by living pulp. On examining it with a low power, the large mass of secondary dentine was found not only to project a good way above the general level of the fractured surface, but also to have, so to speak, overflowed it all round the orifice of the pulp-cavity, and to be everywhere closely adherent to—indeed, continuous with—the old dentine. Roughly speaking, its structure may be thus described: its free or upper surface presented distinct lamination parallel with the surface; next came irregular lacunal spaces; then sparse dentinal tubes; and, finally, in its deepest portion—which was inside the original pulp-cavity—abundant dentinal tubes, which were in places continuous, though oftentimes joining by an abrupt bend, with the dentinal tubes of the original dentine. The overflow onto the fractured upper surface of the original tooth in places was slight, in other places it extended in a gradually thinning layer out to the very edge of the tooth; but what was especially noteworthy was that there were included in the new calcified growth quite a number of entirely detached and displaced splinters of the old dentine.

To proceed to a somewhat more detailed description of the new tissue.

*The Laminated Outer Layer.*—This consists of laminæ parallel with the surface, and varies in thickness, reaching in places $\frac{1}{250}$ inch, and containing about ten well-marked layers. Here and there it constitutes the whole of the overflow, and it contains some canaliculi, taking a direction perpendicular to the surface, and a few well-formed lacunæ with their canaliculi. It is present everywhere, though its amount and the distinctness of the lamination are variable.

*The Lacunal and Interglobular Spaces.*—The tissue immediately below the laminated layer is characterized by an immense number of lacunæ and interglobular spaces, which are in parts well formed and in other parts very coarse and irregular.

The fine boundary of this region of lacunal spaces is in places well defined, and terminates with bodies of the encapsuled lacuna type; elsewhere it passes insensibly into the region occupied by tubes, in the outer part of which latter region interglobular spaces are abundant, and are somewhat irregularly disposed.

*The Tube System.*—In the centre of the tooth the tubes, like those of normal dentine, run vertically upward towards the surface, while towards the sides of the new mass they radiate outward, passing thus beyond the limits of the fractured old dentine, and spreading themselves fan-like over the edges of the original tooth to a certain extent.

In that portion of the secondary dentine which lies within the old dentine (which latter constituted originally the lateral walls of the pulp-cavity) the tubes run more or less outward, and are joined up into continuity with the old dentinal tube, there being generally an abrupt bend and some dilatation at the junction.

It will thus be seen that the whole boundary of the resultant pulp-cavity, formed at its sides and below by the original dentine and above by the new secondary dentine, is formed of dentinal tubes of normal appearance, and that the pulp, though diminished in bulk, has almost perfectly normal surroundings over nearly its whole area.

As the tubes run outward they become more widely separated, owing to their fan-like spreading; it is noteworthy that there are not a greater number of tubes in the expanded portion, but that the interstices between them become larger. A good many short lateral branches are given off, such as those which occur abundantly in the dentine of the roots of normal teeth.

Towards their outer extremities many of the tubes show longitudinal dilatations, and are joined up to the canaliculi of lacunal spaces; some end in brush-like expansions, while others terminate in loops, the loops being common to two or more tubes; others are sharply bent back on themselves. At and above the ends of the tubes fine globular formations may here and there be very distinctly seen.

*The Included Splinters of Dentine.*—As has already been mentioned, this specimen is probably unique, in that the secondary dentine mass contains quite a number of little detached pieces of the original dentine of the tooth which were splintered off in the original attempt at extraction, and which have become solidly inclosed in the new formation.

They have been displaced in various ways, so that their tube systems run in all sorts of directions, and are in no way conformable with the tubes of the new growth. But they have, in their irregularity of position, this much in common, that the tubes of the new growth, when they are of any size, do not pass beyond them, but terminate beneath them. To this, however, there are some exceptions, where quite small chips appear to have been driven more deeply into the pulp.

Upon the whole, then, it may be said that the broken fragments of old dentine either lie embedded in the region of lacunal spaces, or between this and the commencement of the tube system. It is not a little remarkable that none of the fragments show the least sign of absorption, but that their edges are left quite angular, just as they were broken off. Where the tubes commence close against the fragments, they are bent about, obviously with relation to the included pieces.

*Marks of Absorption.*—It is notable that notwithstanding the violent irritation to which the pulp was subject, in very few places can any mark of absorption be found.

The occurrence of "encapsuled lacuna"-like forms has already been mentioned where the lacunal region merges into that of well-formed tubes, but a few marks of absorption and subsequent calcification are to be found elsewhere, and in unlikely places. Thus under the calcified overflow are some pits occupied by Howship's lacunæ.

Every one must, I think, agree with me in astonishment at the extraordinary vitality of this pulp and its amazing success in repairing damages, and it is worth while to examine, or at least to speculate, upon the conditions under which this took place. The whole roof and a little of the sides of the pulp-cavity had been torn off, and the pulp thus widely exposed, apparently a little below the edge of the gum. This must have been temporarily protected by the formation of a coagulum, and ultimately by the contraction of the edges of the gum and its almost complete healing over it, and under these conditions its extraordinarily successful calcification went on.

Is there not a practical hint to be derived from this? Here was a lacerated pulp with loose fragments of sharp splintered dentine jammed into it, coated over only with coagulum, and it did not die nor inflame, but calcified. I think that in capping a pulp, and especially a traumatic exposure, we should probably do better to avoid wiping away any blood or exudation, but leave the effused blood to coagulate; we can put nothing better upon the pulp surface. And probably, when we do commence to cover it, we should do best to put something organic,—sterilized fibrin or gelatin, for instance,—and I shall certainly try such a course of procedure when the opportunity offers, and refrain from placing in contact with the pulp either inorganic materials as a vehicle for medication, or any strong medicaments.

But there is another and less hopeful side to the suggestions presented by this case; there was almost complete success in the formation of secondary dentine, with absolutely no loose nodules or irregular encroachment on the pulp; in fact, precisely the condition which we hope to obtain when a pulp is capped; and yet it is not comfortable, and notwithstanding its full protection under the gum, it became the site of very characteristic pulp-irritation, and consequent neuralgic pain.

Was this an accident? or is the capping of pulps to end in this way usually? Clearly we can hope for no better results in the way of repair; yet why did it become so painful? For all that we can see post mortem, the immediate surroundings of this pulp had become almost exactly those of a healthy pulp, with its dental tubes radiating from it.

Another set of speculations of a more theoretical kind arises. How was the calcification done? Ordinarily the odontoblasts would be torn off and remain adherent to the portion of the tooth which was broken away in the attempted extraction. Were they not torn off, or were they reformed, or was it all done without odontoblasts? If so, then dentinal tubes can be manufactured without odontoblasts, which, from what we know of the process, does not seem likely. But in any case, the first-formed or outer layers are laminated, unlike anything which happens in normal tooth-formation. Were these laminated layers a plastic exudation shed out from the wounded pulp, subsequently organized and finally calcified? I confess that this idea rather commends itself to me, as it would give an easy explanation of the way in which the new tissue flows over the fractured surfaces exactly as if it had got there in a fluid form.

One section seems to afford clinical proof that the material which subsequently calcified was originally fluid. A piece of old dentine has been raised at one end, but left attached at the other, just as happens if a chisel is driven into wood nearly parallel with its surface, but the chip not detached. This has been glued on by something which ran in right under the raised portion with a degree of completeness which strongly suggests its original fluidity.

The same idea is equally strongly suggested by the manner in which the overflow, subsequently calcified, ran out in places over the whole fractured top of the tooth, reaching even to its very outside in a gradually thinning-out layer.

So far as it is possible to read the history of events this appears to have happened. The roof and part of the sides of the pulp-chamber were torn off, and the exposed part, probably retaining its odontoblast layer, swelled out somewhat from the orifice (as indicated by the fan-like expansion of the tubes), and shed out, as it certainly would, plastic exudation over its whole surface, which flowed out over the top of the tooth left. This plastic exudation became permeated by migrating leucocytes, and in and under it the fragments of dentine were inclosed. This was protected by blood-clot, and ultimately by the healing over of the gum, etc. This organized exudation afforded the means for the calcification of the laminated and also of the lacunal tissue, and also for the absorption, and ultimately formation of lacunæ of Howship where this had happened. After the fibrillation and organization of the effused plastic exudation, the pulp itself commenced to calcify in the ordinary way, its odontoblast layer determining the number and form of its tube systems. That this was the case is indicated by the fact that, though the area is larger, there are not more tubes, but only larger interspaces between the tubes, on its expanded portion, and so far as it goes points to there being neither a fresh formation nor multiplication of the odontoblasts. They were stretched apart, and so in the stretched portion the tubes are far apart, becoming dense in the more expanded portion. Thus, so far as it goes, it is a strong confirmation of the view that a dentine tube is a consequence of the presence of an odontoblast. The fragments of dentine, with the exception of a few small pieces which were driven in more deeply, lay on the surface of the pulp, and were stuck to it by the plastic exudation. Hence the dentinal tubes commence under them (with trivial exception), and mark the limits of pulp tissue and of exudation tissue.—*Odontological Society of Great Britain.*

